# Three-dimensional printing and 3D slicer powerful tools in understanding and treating neurosurgical diseases

**DOI:** 10.3389/fsurg.2022.1030081

**Published:** 2022-10-14

**Authors:** Yijie You, Yunlian Niu, Fengbing Sun, Sheng Huang, Peiyuan Ding, Xuhui Wang, Xin Zhang, Jian Zhang

**Affiliations:** ^1^ Department of Neurosurgery, Xinhua Hospital Chongming Branch, Shanghai, China; ^2^ Department of Neurology, Xinhua Hospital Chongming Branch, Shanghai, China; ^3^ Department of Neurosurgery, Xinhua Hospital Affiliated to Shanghai JiaoTong University School of Medicine, The Cranial Nerve Disease Center of Shanghai JiaoTong University, Shanghai, China; ^4^ Educational Administrative Department, Shanghai Chongming Health School, Shanghai, China

**Keywords:** 3D printing, 3D slicer, neurosurgery, additive manufacturing, solid modeling

## Abstract

With the development of the 3D printing industry, clinicians can research 3D printing in preoperative planning, individualized implantable materials manufacturing, and biomedical tissue modeling. Although the increased applications of 3D printing in many surgical disciplines, numerous doctors do not have the specialized range of abilities to utilize this exciting and valuable innovation. Additionally, as the applications of 3D printing technology have increased within the medical field, so have the number of printable materials and 3D printers. Therefore, clinicians need to stay up-to-date on this emerging technology for benefit. However, 3D printing technology relies heavily on 3D design. 3D Slicer can transform medical images into digital models to prepare for 3D printing. Due to most doctors lacking the technical skills to use 3D design and modeling software, we introduced the 3D Slicer to solve this problem. Our goal is to review the history of 3D printing and medical applications in this review. In addition, we summarized 3D Slicer technologies in neurosurgery. We hope this article will enable many clinicians to leverage the power of 3D printing and 3D Slicer.

3D printing allows digital surface models to generate physical models using a printer. It has been used in various medical disciplines, such as plastic surgery, orthopedics, maxillofacial surgery, neurosurgery, and cardiac surgery (1). This article aims to provide an overview of 3D Slicer and 3D printing technology and their applications in neurosurgery. We will introduce how 3D printing and 3D slicers can improve neurosurgery research and practice. We believe it can raise neurosurgeons' awareness to create digital anatomical models to assist their daily practice.

We identified studies published until 27 June 2022 through systematically searching EMBASE, PubMed, Web of Science, Elsevier, Medline, and Cochrane Library. We used the combination of the following terms (3D Slicer, 3D printing, and Neurosurgery) in the title/abstract/keywords or subject terms. We used Boolean operators “AND” or “OR” to combine the literature searches. Finally, we wrote this article *via* careful evaluation.

## Overview of 3D Printing

**Figure 1:**
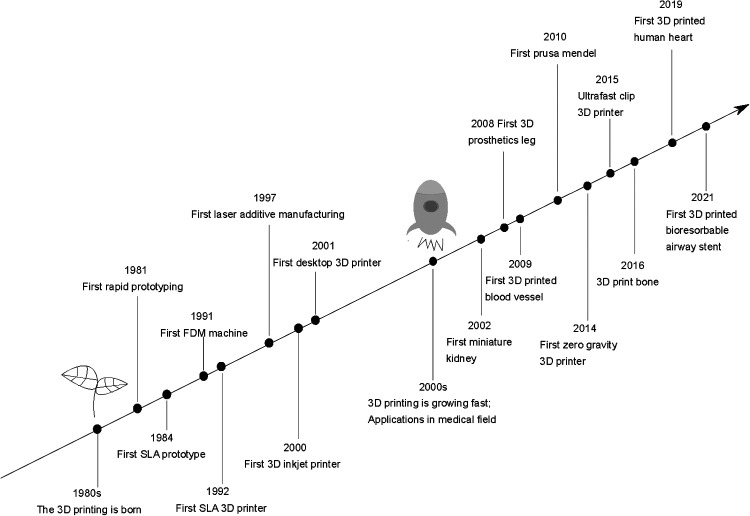
Timeline of 3D printing technologies.

In 1981, Dr. Hideo Kodama first proposed the 3D printing technology *via* fabricating a device that uses ultraviolet lights to harden polymer and create solid objects (2). On this basis, Charles Hull invented the first 3D printer (the stereolithography machine, SLA) in 1983 (3). Then, the selective laser sintering (SLS) technology was developed by Dr. Carl Deckard in 1987. One year later, there was the first rapid prototyping printer entitled SLA-1 appeared in the commercial market. In 1989, Scott Crump invented fused deposition modeling (FDM), popularly used for polymeric materials. Subsequently, other technologies such as inkjet printing, laser AM (LAM) and various subsequent methods were introduced. Today, the 3D printing industry leaders are two famous companies, 3D Systems and Stratasys (4–6). With the development of 3D printing technology, Swiss scientists fabricated customized bioresorbable airway stents with elastomeric properties in 2021 (7). Figure 1 schematically represents the 3D printing history and its achievements in the medical field.

**Figure 2:**
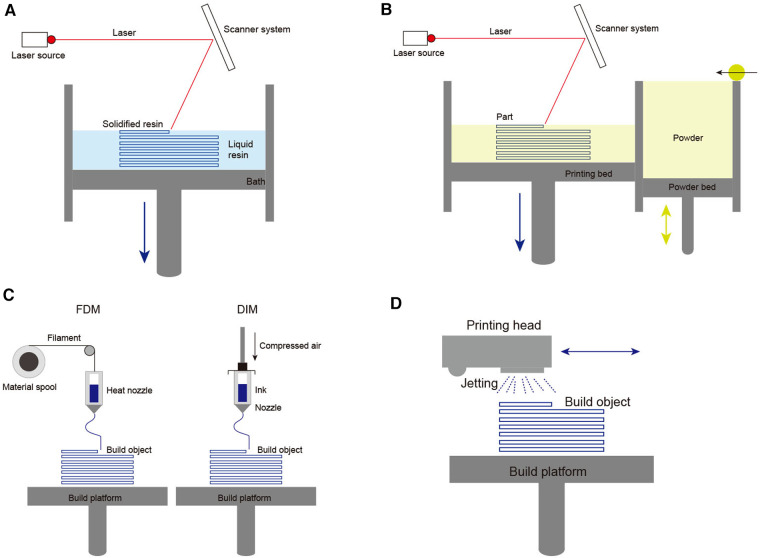
Schematic diagrams of 3D printing technologies main methods: (**A**) Basic principle of vat polymerization-based printing; (**B**) Basic principle of powder-based printing technologies; (**C**) Basic principle of extrusion-based technologies; (**D**) Basic principle of droplet-based printing.

A brief overview of additive manufacturing technologies is necessary to understand better the principles of 3D printing (Figure 2). The most commonly available 3D printing technologies mainly include four groups: vat polymerization-based printing, powder-based printing, extrusion-based printing, and droplet-based printing. First, vat polymerization-based printing uses a specific wavelength laser to locally cure the resin one layer at one time *via* generating an ultraviolet beam at the surface of a vat of photocurable resin. This technology mainly includes four modes: stereolithography (SLA), direct or digital light processing (DLP), and continuous liquid interface production (CDLP). This technology offers high geometric accuracy, but the resin material restricts its' development (Figure 2A) (8). Second, powder-based printing technologies rely on local heating to fuse thermoplastic powder made from plastic, metal, or ceramic. Powder-based printing technologies include selective laser sintering (SLS), direct metal laser sintering (DMLS), electron beam melting (EBM), and selective laser melting (SLM). To generate local heating, SLS, DMLS and SLM use laser beams directed by mirrors, whereas EBM utilizes a high-energy electron beam precisely directed by electromagnetic coils. Then, after local heating fusing a cross-section, the powder bed drops down one layer, and a new layer of thermoplastic powder is created. Despite this method can produce nearly fully dense parts, needing a higher cost and limited material availability (Figure 2B) (9). Third, extrusion-based technologies include fused deposition modeling (FDM) and direct ink writing (DIW). FDM uses thermoplastic or composite filaments (polylactic acid, acrylonitrile butadiene styrene, polyamides, polystyrene, polycarbonate), which are extruded through a hot nozzle. FDM provides high geometric accuracy and models that withstand the disinfection process in an operative setting. DIW utilizes a pneumatic or mechanical dispensing system to extrude concentrated suspensions formulated of primary material together through a nozzle or a syringe. This method is a well-known technique for obtaining ceramic pieces with complex geometry, but it is challenging to acquire highly dense pieces (Figure 2C) (10). Fourth, droplet-based printing is defined as computer-controlled non-contact pattern reproduction techniques, mainly including multi-jet modeling (MJM), wax deposition modeling (WDM), laser-induced forward transfer (LIFT), and binder jetting (BJ). The technology relies on the precise jetting of liquid droplets onto a substrate layer-by-layer manner. Although droplet-based printing allows full-color prototyping, it weakens the mechanical strength (Figure 2D) (11).

##  Printing Materials

3D printing quality depends on the 3D printer and materials. The appropriate material is necessary to obtain a 3D-printed model with optimal properties. 3D printing materials for medical applications include but are not limited to polymers, metals, and ceramics (12). Of these, polymers, like polyether ether ketone or polystyrene, are those most commonly used. Because carbon-based materials are flexible and economical, they can be used to achieve desired properties. Like steel and titanium, metals have a long history of medical applications due to their strength, stiffness, and malleability. However, they tend to be more expensive than polymers and are more vulnerable to corrosion. Ceramics are insulating and resistant to thermal or corrosive degradation. However, ceramics are fragile and are difficult to reshape with their powders. Lastly, composites combine polymers, metals, or ceramics to create a material with respective properties. Today, many 3D printing materials are composites designed to acquire desired strength, flexibility, durability, and cost-effectiveness (13–15). A recent review elaborated on the advances in 3D printing composites (16).

##  Printing and Medical Applications

3D printing technology involves many medical fields, including plastic surgery, medical and pharmaceutical industries, neurosurgery, and orthopedics (17–20). As the technology develops, tissue and organ printing is an emerging field that has proved valuable in surgical planning, anatomic prostheses, and trainee education (21, 22). In addition, 3D printing has become integral to pharmaceuticals. This way, drugs can be individually printed in specified doses, and the speed of drug delivery can be controlled (23, 24). What also deserves expecting part of the technology is potential applications of 3D biological printing, which have already hinted at the feasibility of biological printing artificial organs *via* the dispensing of cell-laden hydrogels (25).

##  Printing in Neurosurgery

In neurosurgery, it is hard to observe outwardly the most surgical procedures involving intricate, minute anatomical structures. Neurosurgeons rely on neuroimaging to prepare for highly customized procedures (26). Standard imaging methods include x-ray, CT, and MRI (27, 28). However, it is difficult to appreciate the 3D relations between these structures within a limited surgical area. With 3D printing technology, clinicians could acquire an effective solution. 3D printing translates anatomical structures into 3D images and fabricates physical models for preoperative planning and practice education. 3D printed materials can also be applied to the design of surgical simulations. The simulations provide a realistic scene to help surgeons rehearse operations as often as desired, reducing patients' potential harm risk (29–31). Moreover, 3D printing can be used for prototyping and producing innovative surgical devices, just like its' function in the manufacturing industry. This technology can create instruments and implants corresponding to individual patient anatomy to achieve a personalized treatment (32, 33). In research scenarios, 3D printed models can replace animals and human bodies to explore the biophysical characteristics of different conditions on tissues (34, 35). In summary, 3D printing in neurosurgery has four main areas:

1. Creating anatomical models for surgical planning, training, and education.
2. The invention of neurosurgical devices for treating and assessing neurosurgical diseases.
3. The research and development of biological tissue-engineered implants.
4. The research of tissues for biophysical characteristics.

Several articles have reported 3D printing in neurosurgery. For example, Kondo et al. generated rapid prototyping models of heads with unruptured cerebral aneurysms for 22 patients. The models perfectly reproduced the microsurgical anatomy and arteries (36). Later, Namba et al. successfully predetermined the optimal shape before the endovascular surgery *via* the 3D printer aneurysm model (37). In addition, printed head models were used to plan and develop new treatments for brain tumors. Makoto et al. successfully produced 3D printed plaster models of the brain to determine the optimal surgical strategies for skull base and deep tumors. Although there are some unpredicted problems, such as severe bleeding or tumor tissue hardness, surgeons could quickly solve the problems using a planned optimal surgical window (38). Moreover, 3D printing has proven to be a valuable tool in neurosurgical devices. 3D printing enables researchers to create printed devices for recording brain activity in noninvasive forms. For example, Troebinger et al. reduced between and within-session coregistration errors in Magnetoencephalography *via* 3D printed subject-specific head-casts (39). Furthermore, 3D printing could create a mold of the decompressed segment of the skull to perform cranioplasty. Many researchers used the mold as an implant created from many materials, such as titanium, acrylic, and polyether ketone (40–45). For example, Rok Cho et al. achieved cranioplasty *via* 3D printed porous titanium implants. The 3D-printed implants perfectly repaired skull defects without specific complications and dead space (46). Similarly, advances in 3D printing technology have revolutionized the treatment of craniosynostosis. Several articles have demonstrated the potential benefits of 3D printing in craniosynostosis, such as simplifying surgical procedures and specifying personalized templates. However, the literature is still in the validation stage, and more extensive case-series studies are needed to illustrate this approach's efficacy further. Modeling techniques, implant cost, and long-term skull fitness must also be improved (47). In scientific research, Kolli et al. used 3D printed models to research the effect of varying hemodynamic conditions on fractional flow reserve. The result shows that increased aortic pressure reduced fractional flow reserve value in stenosis for vascular groups with or without myocardial infarction (34). These articles, when taken together, represent just a part of aspects. With the fast development of the technologies, more and more novel applications of 3D printing have come out in practice (31).

## Limitations in 3D Medical Printing

There are several limitations to the widespread application of 3D design and printing in neurosurgery:

1. There is a speed limitation. Because printers need a long time to build 3D models, some acute diseases such as intracerebral hemorrhage have no time to wait to achieve 3D models (48). Although some companies claim that their printer combines the benefits of good printing speed and high resolution, the practicability needs to be verified in further clinical studies (49).
2. Material is another kind of limitation. There are no particular standards on the international to select medical materials for 3D printing. Thus, doctors only choose materials in structure, function, clinical effects, and clinical experience rather than choices based on reliable indicators and experimental evidence (50).
3. The cost of 3D printing is uncertain. The cost of 3D printed parts depends heavily on the manufacturing facilities and materials. Although various cheap desktop 3D printers exist in the market, these printers are difficult to achieve high-quality standards. However, some high-end printers need special people trained by a customer support representative to operate them. If human resource costs, time costs, and material costs are included, the total cost of 3D printing will be huge. In addition, materials and filaments are consumables. Exorbitant prices and short expiry dates increase the costs of 3D printers, especially for high-end printers (1, 31).
4. Many surgeons lack the technical skillset of 3D designing and modeling. This lack results in slower adoption of 3D printing in the medical field. So we hope to surmount this barrier by demonstrating the use of 3D Slicer for digital modeling.

## Introduction of 3D Slicer

3D Slicer, a multiplatform software that runs on personal computers, can directly visualize the patients' anatomy with various imaging techniques (51). It enables the fusion of anatomical data and functions and provides various generic and specialized tools for processing and multimodal analysis (Table 1). David Gering first proposed the prototype of Slicer in his master's thesis in 1999, based on the experience of earlier research groups at the Massachusetts Institute of Technology and the surgical planning laboratory of the Brigham and Women's Hospital in Boston. Subsequently, Steve Pieper served as the lead architect of Slicer, commercializing the 3D Slicer to meet the requirements of industrial-grade installation packages. The development of 3D Slicer has been followed since 1999 by the Surgical Planning Laboratory, led by Ron Kikinis. Today, 3D Slicer is a collaborative effort of professional engineers, algorithm developers, and applied scientists. Companies like Isomics, Kitware, and GE Global Research have joined Slicer. The growing Slicer community has also contributed significantly to its development. 3D Slicer was initially conceived as a system for guided treatment, visualization, and analysis in neurosurgery. However, over the decades, 3D Slicer has evolved into a comprehensive platform that can be used for various clinical and preclinical research applications and non-medical image analysis (52).

**Table 1:** Commonly available digital visualization programs.

Program	Platform	Plug-ins	Memory (bit)	Cost	URL
3D Slicer	Mac OS, Windows, Linux	Yes	64	Free	https://www.slicer.org/
Horos	Mac OS	Yes	64	Free	https://www.horosproject.org/
OsiriX	Mac OS	Yes	32 or 64	Free or $69.9	https://www.osirix-viewer.com/
Mimics	Windows	No	64	Variable	https://www.materialise.com/en/medical/mimics-innovation-suite
MediPy	Windows, Mac OS, Linux	Yes	64	Free	https://code.google.com/*p*/medipy/
WEASIS	Windows, Linux, Mac OS	No	32 or 64	Free	https://nroduit.github.io/en/
MITK	Windows, Mac OS, Linux	Yes	64	Free	https://www.mitk.org/wiki/The_Medical_Imaging_Interaction_Toolkit_ (MITK)
ITK-SNAP	Windows, Mac OS, Linux	Yes	32 or 64	Free	http://www.itksnap.org/pmwiki/pmwiki.php
ParaView	Windows, Mac OS, Linux	Yes	64	Free	https://www.paraview.org/
VTK	Windows, Mac OS, Linux	Yes	64	Free	https://vtk.org/

**Figure 3:**
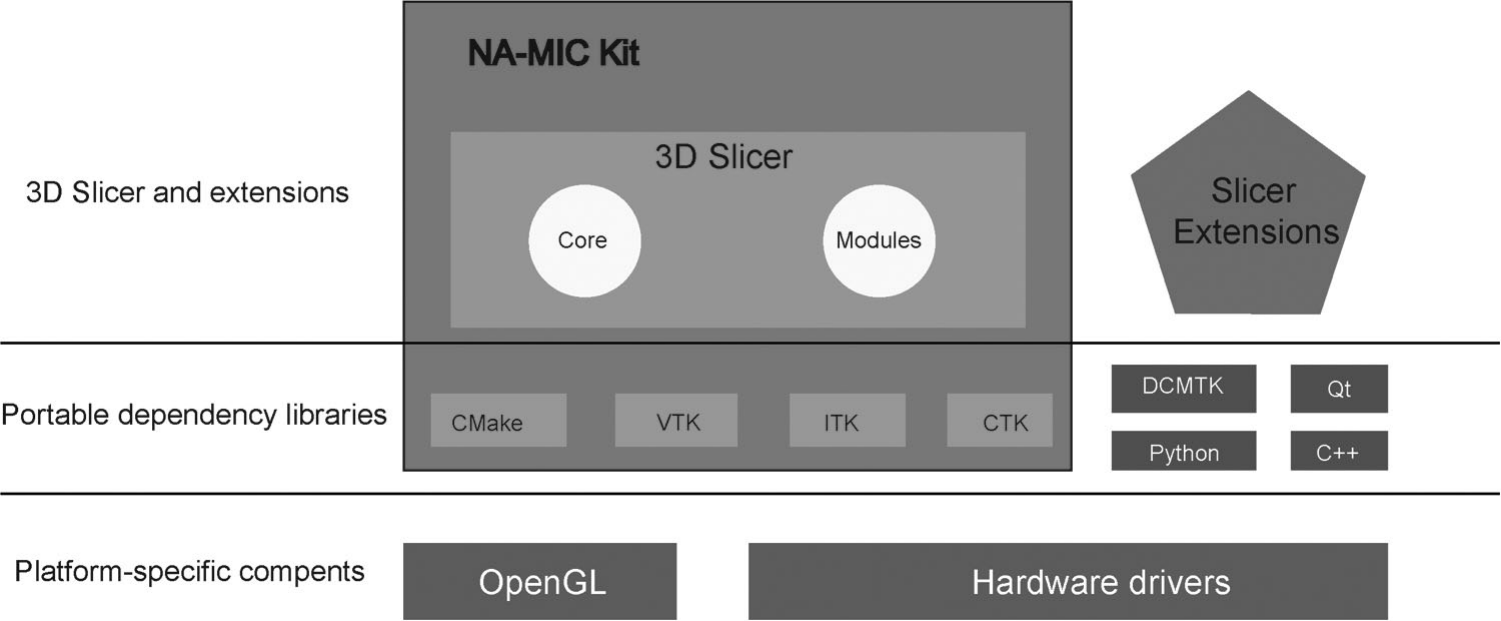
3D slicer ecosystem. 3D Slicer consists of the lean application core, Slicer Modules, and Slicer Extensions. Languages (primarily C++, Python, and JavaScript) and library (Qt) provide higher-level functionality and abstractions. NA-MIC Kit components provide the tools and interfaces for the needs of the developers of medical image computing applications. CMake enables cross-platform build system configuration, packaging, testing of 3D Slicer and NA-MIC Kit libraries. VTK (Visualization Toolkit) provides the key building blocks for 3D computer graphics and visualization. ITK (The Insight Toolkit) is a library developed specifically for the tasks related to medical image registration and segmentation, and for implementing new image analysis algorithms. CTK (Common Toolkit) is a biomedical image computing library with a focus on application-level DICOM support, plugin framework, and specialized GUI widgets.

The architecture of 3D Slicer follows a modular and layered approach (Figure 3). The virtual libraries such as OpenGL and hardware drivers are not packaged with 3D Slicer and are not provided by the operating system at the lower architecture level. At the level above, there are languages (primarily C++, Python, and JavaScript) and libraries (Qt, DCMTK, jqPlot) that provide higher-level functionality and abstractions (53, 54). All the external dependencies of 3D Slicer are cross-platform portable distributed under licenses fully compatible with 3D Slicer, which made it widely used in commercial or open-source products. The great advantage of 3D Slicer is its versatility because it provides a robust API (Application programming interface) for interacting with the software using self-made code in python and other programming languages. This point enables users to build new tools and applications on top of 3D Slicer.

As a free open source extensible software, the basic capabilities of 3D Slicer include visualization, registration, segmentation, and quantification of medical data (55). Moreover, 3D Slicer can be extended to enable the development of interactive and batch processing tools for various applications (56). For example, through external code, 3D Slicer enables various applications, including fiber tract, quantification, radiomics, and AR. The development of 3D Slicer cannot be separated from a community effort. Since many groups and individual users are continually improving 3D Slicer without pay through reporting software issues and providing solutions, suggesting new features, and developing new express tools.

A few decades ago, 3D Slicer was used in computer-assisted 3D planning, navigation, and intra-operative imaging (51, 57–60). In recent years, neurosurgical trends have been minimal invasiveness and maximal safety (61). However, many tools, such as neurosurgery navigation systems and intraoperative imaging techniques, tend to be expensive and thus difficult to implement in low and middle-income areas (62–64). 3D Slicer has the characteristics of novelty, easy operation, and low cost, and it can be used for diagnosis and pre-surgical planning in neurosurgery (51, 52). Some new ideas can be provided for surgeons to develop better operative procedures *via* combining 3D Slicer with 3D printing in neurosurgery.

## Use of 3D Slicer in Neurosurgery Studies

### 3D slicer and 3D reconstruction

3D Slicer can enable new algorithms and applications by extending this software. It allows multiple configurations, from a simple 3D visualization of medical images to different reconstructions and generating images (65). In neurosurgery, doctors use traditional 3D technology such as MRI (Magnetic Resonant Imaging) or CT (Computed Tomography) scans of the brain to view the lesion locations. An automatic system of 3D Slicer can be used for segmentation analyzed in 3D, giving a complete view to the doctors of the size and position of lesions in the brain. For example, 3D Slicer is usually used in reconstructing the complete views of glioma, cranial nerves, intraventricular, blood vessel, and intracerebral hematoma (66–69).

### 3D slicer and brain tumor

Glioma is the most aggressive form of brain tumor in adults. The main treatments include surgery, radiotherapy, and chemotherapy (70). Because the resection area has an essential effect on prognosis, determining the tumor boundary and detecting the resection area is significant (71). 3D Slicer can provide a robust, easy-to-use image informatics framework that avails interactive segmentation of brain tumors and image-guided therapy (72). For example, Liang et al. prepared a pre-operative plan with 3D Slicer software based on pre-operative MRI and obtained a maximum extent of glioma resection and functional protection (73). However, the accuracy of surgery is reduced if the pre-operative plan is based only on initial MR images. Because of brain shift and brain tissue deformation, this results in an actual anatomical location different from the virtual one. Although some researchers have proposed that surgeons can use intraoperative MRI and 3D Slicer to solve brain shift and brain tissue deformation (51, 60, 74), we are still waiting for a more straightforward method that relies on 3D Slicer.

### 3D slicer and cranial nerve

With recent advances in modern neuroimaging, more and more cranial nerves can be detected *via* medical imaging. Preservation of cranial nerve function remains an important goal to resect brain lesions. In recent years, as 3D Slicer developed, visualization of the 3-dimensional anatomy of brain lesions with their surrounding cranial nerve can be reconstructed, improving the safety and validity of operation by drawing up a preoperative plan to protect critical neural structures (66, 67). Jun et al. verified that the 3D Slicer technique could accurately show the encasing and neighboring relationship between nerves and intracranial lesions *via* intraoperative exploration (75). Especially, 3D Slicer provides high-quality 3D visualization which shows the relationships between nerves and blood vessels in patients with trigeminal neuralgia. That performs a preoperative evaluation for microvascular decompression in clinical practice (76, 77). However, due to the limited number of samples, this technology needs more studies with relevant statistical analysis to assess the clinical effect.

### 3D slicer and fiber tract

Knowledge of the anatomy of fiber tracts becomes essential when neurosurgeons begin to plan tumor resections (78). Surgeons must avoid severe impairment in patients' motor, cognitive, or visual functions, so the integrality of fiber tracts should remain (79, 80). Multiple international research centers have developed recent applications of 3D Slicer in neurosurgery and oncology. It has many diffusion functions, such as fiber tractography, fiber selection, and fiber reconstruction (81). Actually, *via* 3D Slicer, Yang et al. successfully used the self-constructed 3D virtual images to achieve neurosurgical operations, avoiding damaging fiber bundles as much as possible (27). 3D Slicer can use the plugin “MultiXplore” to achieve cerebral fiber imaging. Unlike traditional connectivity visualization technology, 3D Slicer relies on the plugin “Multixplore” to display more realistic graphics and help doctors associate nerve fiber bundles with anatomical structures. Bakhshmand et al. introduced in detail how to use the MultiXplore (82).

### 3D slicer and quantification

Neurosurgeons can use 3D Slicer to calculate the volumetric changes for evaluating many clinical issues. For example, Li et al. accessed the effectiveness of neuro endoscopy application in treating middle cranial fossa arachnoid cysts *via* 3D Slicer reconstruction and quantitative calculation (83). Xu et al. found that 3D Slicer is a low-cost, accurate, and practical technique for measuring intracerebral hematoma volume (84). Cheng et al. used 3D Slicer to measure and compare the volume of the cisternal segment of trigeminal nerves to research whether nerve atrophy affected the efficacy of microscopic vascular decompression. Volumetric measurements of 3D Slicer software were also used in assessing the relationship between tumor growth rates and the histological grade (85). Furthermore, volumetric segmentation of 3D Slicer demonstrated predictive value in cerebrospinal fluid leaks and correlated with postoperative complications (86). We expect more new applications to predict other unknown territories.

### 3D slicer and radiomics

Currently, radiomics is widely used in central nervous system diseases. The core hypothesis of “radiomics’ is that many image features are extracted from radiological images and then transformed into exploitable feature space data that can display the characteristics of diseases (87, 88). Because 3D Slicer software can be used to calculate the radiomics features, it has extensive use in clinical research. Cellina et al. found that radiomics analyses extracted from the optic nerve by 3D Slicer can assess the visual function and predict multiple sclerosis development (89). Additionally, Qi et al. explored the feasibility of predicting early-stage brain cognitive impairment through radiomics (90). Combining 3D Slicer and radiological metrics as a new research direction has enormous potential in disease diagnosis and recognition.

### 3D slicer and augmented reality (ar) technology

Augmented reality is a recently developed technology that adds information to the actual surgical field through computers (91). In many developing countries, 3D rendering software such as 3D Slicer, NIRFast, or ITK-Snap running on a computer forms primary AR systems (92, 93). In neurosurgery, augmented reality is currently available for simulation and training. However, using AR in the clinic is in its infancy because of the barriers and limitations of the technology (91). Inoue et al. utilize AR technology and 3D Slicer to develop a neuronavigation system for helping surgeons to perform safe surgical procedures. The navigation system needs to be further optimized due to the limitation of precise measurement and eye movement (94). Even so, we believe that AR technology and 3D Slicer can hold a bright prospect and a significant potential for application in neurosurgery.

##  Slicer and 3D printing

Three-dimensional printing (3D printing), specific additive manufacturing, is an inexpensive and accessible fabrication technique for transforming digital objects into physical models (95). Digital models were processed using 3D Slicer software to prepare for the 3D printing. The lifelike models created by 3D printing have an obvious advantage in training and surgical planning (96). Similarly, other advantages of 3D printing can be achieved, such as researching and developing biological tissue-engineered implants.

**Figure 4:**
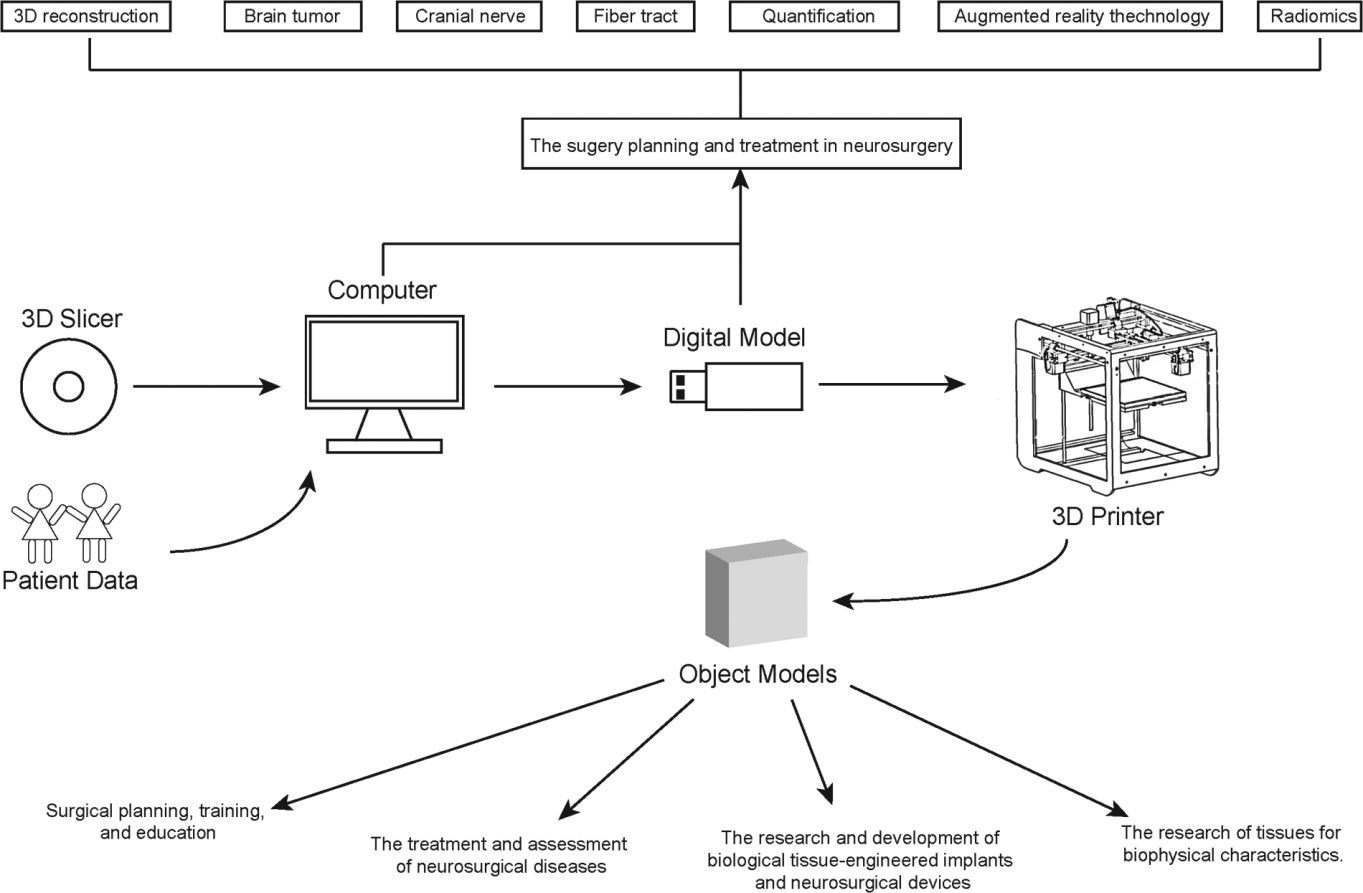
3D printing and 3D slicer in understanding and treating neurosurgical diseases.

Many research articles have made use of 3D Slicer and 3D printing. For example, Memon et al. used 3D Slicer to segment the descending aorta, renal artery, and renal anatomy to create a computer-aided image for 3D printed models. They found that pre-operative 3D printing of renal artery anatomy could help medical staff reduce contrast, fluoroscopy, and procedure time (97). Moser et al. used 3D Slicer and 3D printer to prefabricate osteosynthesis plates for maxillofacial surgery (98). Cheng et al. used 3D Slicer to design a personalized airway prosthesis and then printed it *via* a 3D printer (99). Xu et al. used 3D Slicer to obtain 3D airway models. Stent customizations were made based on the 3D model dimensions *via* 3D printing. After the printed stents were inserted, they found improved patient airway performance (100). In neurosurgery, 3D printing has been applied in skull base procedures and trauma, such as 3D printing navigation models and skull reconstruction (101–103). These examples are just the tip of the iceberg, but they demonstrate a successful combination between 3D Slicer and 3D printing in medicine. We specially design a picture to show how 3D printing and 3D slicer can improve neurosurgery research and practice (Figure 4).

### Limitation

Some neurosurgical diseases are acute, such as acute cerebral hemorrhage and cerebral hernia. Using 3D Slicer and 3D printing for preoperative planning may delay the treatment of these diseases. Patients will miss the best operation timing if a doctor accomplishes a modeling process that includes data collection, data processing, and model work. Moreover, because most doctors specialize in medicine or operation, studying 3D Slicer and 3D printing increases their workload. That makes this technology spread slowly in clinical. In addition, due to human resource costs, time costs, and material costs, the cost of most 3D printing is enormous, which will make Low- and Middle- income countries challenging to afford. However, simple operation training may be achieved if low-cost printers and materials are selected. For example, Low- and Middle-income areas choose polyester fiber for printing 3D models, which is rough but enough for neurosurgical training and practice. As for 3D Slicer, we believe it is suitable for pre-operation planning due to its free in Low- and Middle-income areas. In summary, 3D Slicer and 3D printing can become powerful medical tools in economically undeveloped areas.

## Three-dimensional Printing and 3D Slicer Powerful Tools in Neurosurgery

Because of the above summary of 3D Slicer and 3D printing, the knowledge displayed so far is relatively professional and abstract. We specifically summarized relevant examples to increase neurosurgeons' interest and further illustrated the application of 3D Slicer and 3D printing in neurosurgery. At the same time, we drawn related videos to demonstrate the essential software operation. We hope to provide readers with an accessible introduction to the operation *via* these videos. It is important to note that these videos do not show the latest graphics techniques. For more advanced graphics and modeling techniques, refer to the experience shared in the 3D Slicer forum (https://www.slicer.org/). Because our paper focused on combining 3D Slicer and 3D printing in neurosurgery, we only summarized the relevant example of 3D Slicer software here. It should be noted that many software on the market can replace 3D Slicer to carry out modeling. Readers can draw inferentially from this point and use 3d printing to conduct more relevant research.

### Preoperative planning in clipping of intracranial aneurysms

Maciej et al. realized preoperative planning of intracranial aneurysms through combining 3D Slicer and 3D printing, achieving a low-cost and high-precision surgical effect. Moreover, their research further proved that combining these two technologies can achieve good preoperative planning and clinical teaching significance in neurosurgery. Their main step was to collect imaging data and then use 3D Slicer software to build a G-code file that a printer could read to print out a 3D physical model. Finally, the physical model was used for preoperative planning and clinical teaching (104).

### Protective cap in brain protection after decompressive craniectomy

Based on brain CT data, Shi et al. designed a brain-protective cap device using 3D Slicer and 3D printing technology. The device can protect patients with skull defects from brain tissue compression, and its clinical results show specific clinical value, which can be used to improve the quality of life of patients who cannot receive skull repair. In their study, the CT data of patients after surgery was processed by 3D Slicer, and the defects were completed in 3D modeling to form a whole cranial model. Then the defective skull data was selected to form the defective skull model. Finally, the defect model data was put into a 3D printer to print out to generate a personalized protective cap. The brain protective cap was fixed to the patient's head by a reticular elastic cap when the patient wore it. In the later follow-up study, they found that the personalized protective cap has the advantages of low price, simple design, and fast. In addition, it can reduce the safety hidden dangers, reduce the incidence of secondary accidental brain injury, improve the skull shape and appearance, and increase the life confidence of patients, which has specific clinical promotion significance (105).

### 3d Printed spine phantoms for biomechanical research

3D Slicer and 3D printing are not only used for clinical research but also for biological research. The new achievements in basic research are also of great help to the subsequent clinical application of neurosurgery. William et al. successfully used 3D Slier to process imaging data and generate 3D cervical spine models and then used 3D printers to print virtual models into physical models to understand the biological functions of cervical vertebrae in different individuals (106). This research is actually of some research value in neurosurgery. Just like spinal canal injury, degenerative or traumatic lesions, such as ossification of the posterior longitudinal ligament, facet hypertrophy, vertebral fracture or dislocation, and many other conditions, can be evaluated by this 3D printed model. Before the surgical intervention, the model allows analysis of patient-specific conditions, such as luminal changes during surgical positioning and dimensional changes after decompression.

### Navigation system technique is used for deeply brain tumor

Moneer et al. used 3D slice software to build 3D models of patients' deep brain tumors. Doctors then determined the trajectory by combining points on the cortex, on the surface of the tumor, and a few millimeters outside the cortex. A 3D printed model was then used to test the accuracy of the surgical approach. For preoperative planning, the doctor identified the entry point through the model and matched it to the one displayed in the software, then pulled the pen mouse up a few millimeters on the sliding trajectory and rotated it until it conformed to a predefined trajectory. Clinical results show good surgical outcomes, with less cost and time than traditional surgical navigation (107). This example perfectly demonstrates the application of 3D Slicer and 3D printing in brain tumor resection. The detailed process can be found in the research of Moneer et al., which provides a detailed video explanation.

### 3D slicer and 3D printer to generate deficient skull models

**Figure 5:**
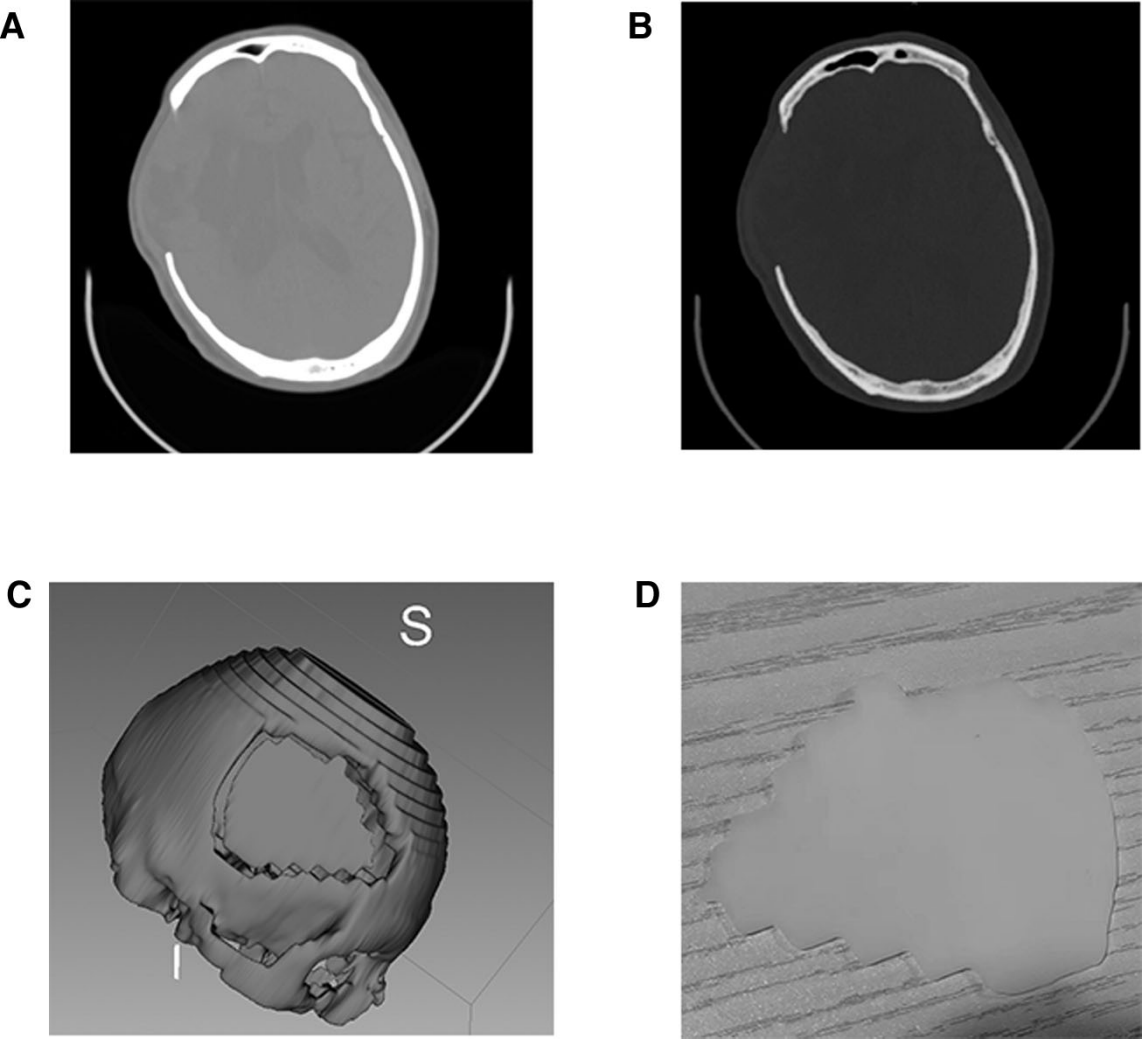
3D printed skull and mold from high resolution CT scan data. (**A,B**) High resolution CT scan data; (**C**) virtual mold in 3D Slicer; (**D**) 3D printed skull.

In our paper, to further demonstrate the related design process more clearly, and to facilitate neurosurgeons to understand the combination of these two techniques further, we take skull repair as an example to make a simple demonstration. It is straightforward to generate a deficient cranial model. After downloading and installing 3D Slicer, the user will need to install the extension for the segment editor. The user will then need to load the CT scan and begin the skull reconstruction. The custom 3D modeling method is used for implant development (Video 1). Once the digital model of the deficient skull model has been generated, the user can save the file into STL or OBJ format, which can then be sent to a 3D printer for printing (Figure 5; Video 2).

## Conclusions and Future Considerations

3D Slicer can assist neurosurgery, and it is currently believed that 3D Slicer provides a low-cost and simple method to improve the efficacy and safety of surgery. Although 3D Slicer is made available for free, any use, such as clinical use, is entirely the user's responsibility, and that local regulation must be observed. On the other hand, 3D Slicer combines with other emerging technologies, such as AR technology or 3D printing to promote the development of minimally invasive neurosurgery. Therefore, it is essential to develop the new capabilities of 3D Slicer, especially in developing countries. After all, frontier technologies (e.g., surgical robots, high precision stereotaxic, and neuro-navigation) will not be utilized in all areas due to economic and scientific levels differences.

As for 3D printing, many challenges remain with the rapid development of 3D printing and increasing medical applications. In current neurosurgery, speed, materials, and uncertain cost restrict the 3D printing to the experiments stage. However, we think there must be an adequate solution to this problem in the future. Because people inevitably discover ideal materials with the development of science and technology. As a novel technology in manufacturing and design, 3D printing could change the future of medicine.

In the current study, the combination of 3D Slicer and 3D printing is still in the research phase. Regrettably, most medical reports on 3D printing and 3D Slicer are experimental studies. More clinical studies and experiments are needed to acquire evidence on the utility and accuracy of these techniques. We hope this review can arouse the attention of medical staff to study 3D Slicer and 3D printing and explore more new applications, areas of cooperation, and new growth points.
